# Microalgal biofilm induces larval settlement in the model marine worm *Platynereis dumerilii*

**DOI:** 10.1098/rsos.240274

**Published:** 2024-09-18

**Authors:** Cameron Hird, Gáspár Jékely, Elizabeth A. Williams

**Affiliations:** ^1^ Scymaris Ltd, Brixham Laboratory, Freshwater Quarry, Brixham, Devon TQ5 8BA, UK; ^2^ University of Exeter, Biosciences, Faculty of Health and Life Sciences, Streatham Campus, Exeter EX4 4QD, UK; ^3^ University of Heidelberg, Centre for Organismal Studies, Im Neuenheimer Feld 230, Heidelberg 69120, Germany; ^4^ University of Exeter Living Systems Institute, Streatham Campus, Exeter EX4 4QD, UK

**Keywords:** *Platynereis*, larval settlement, diatoms, biofilm, microalgae

## Abstract

A free-swimming larval stage features in many marine invertebrate life cycles. To transition to a seafloor-dwelling juvenile stage, larvae need to settle out of the plankton, guided by specific environmental cues that lead them to an ideal habitat for their future life on the seafloor. Although the marine annelid *Platynereis dumerilii* has been cultured in research laboratories since the 1950s and has a free-swimming larval stage, specific environmental cues that induce settlement in this nereid worm are yet to be identified. Here, we demonstrate that microalgal biofilm is a key settlement cue for *P. dumerilii* larvae, inducing earlier onset of settlement and enhancing subsequent juvenile growth as a primary food source. We tested the settlement response of *P. dumerilii* to 40 different strains of microalgae, predominantly diatom species, finding that *P. dumerilii* have species-specific preferences in their choice of settlement substrate. The most effective diatom species for inducing *P. dumerilii* larval settlement were benthic pennate species including *Grammatophora marina*, *Achnanthes brevipes* and *Nitzschia ovalis*. The identification of specific environmental cues for *P. dumerilii* settlement enables a link between its ecology and the sensory and nervous system signalling that regulates larval behaviour and development. Incorporation of diatoms into *P. dumerilii* culture practices will improve the husbandry of this marine invertebrate model.

## 1. Introduction

Numerous marine invertebrates, from sponges to tunicates, face a crucial decision early in their life cycle—as a microscopic planktonic larva, they must select a suitable site on the seafloor on which to settle down, undergo metamorphosis from larval to juvenile form and transition to a benthic, or bottom-dwelling, lifestyle. This major life cycle transition is guided by cues from the surrounding environment, which may attract or repel larvae from particular locations. Common cues that can induce settlement and metamorphosis in marine invertebrates include the presence of conspecifics, microbial biofilms and potential food sources [1]. An improved understanding of the process of larval settlement and metamorphosis is required, as this stage acts as a bottleneck in the life cycle and the process can shape adult marine invertebrate communities, in both aquaculture and biofouling scenarios, as well as in natural marine ecosystems predominated by invertebrates, such as coral reefs [2–4]. Due to the diversity of invertebrate species and potential cues, as well as the challenge of studying microscopic interactions that occur in a macroscopic, three-dimensional environment, we still lack a comprehensive picture of the process of larval settlement, in terms of specific cue detection, the sensory modalities triggered within larvae and the downstream internal signalling pathways that must be activated within a larva in order to drive metamorphosis, for any species.

The marine polychaete, *Platynereis dumerilii*, presents a model with good potential for gaining insight into the larval settlement process. *Platynereis dumerilii* has been established as a laboratory model for several aspects of biology, including neurobiology, development and evolution [5]. *Platynereis dumerilii* represents the relatively understudied phylum Lophotrochozoa, which also includes molluscs, bryozoans, brachiopods and platyhelminthes. Like many marine invertebrates, *P. dumerilii* has a life cycle that includes a planktonic larval stage that undergoes settlement and metamorphosis into a benthic juvenile. Understanding of sensory signalling processes in *P. dumerilli* larvae is greatly facilitated by molecular resources including extensive gene expression atlases in larval stages [6–11], a 3-day-old full-body larval connectome [12] and knowledge of several nervous system signalling molecules and their receptors [13–15]. In addition, genome editing tools have been successfully established in *P. dumerilii*, enabling the study of specific gene functions through either gene knockout or knockdown [16,17].

Despite several decades of laboratory study, it remains unclear whether specific environmental settlement cues exist for *P. dumerilii* larvae. In the natural environment, *P. dumerilii*, which are common in Mediterranean seas, but also distributed throughout European seas, are found in association with seagrass or macroalgae beds [5]. However, *P. dumerilii* can be maintained in a laboratory environment throughout its life cycle, as long as juvenile and tube-dwelling adult stages are fed. The laboratory diet of *P. dumerilii* commonly consists of green microalgae, such as *Tetraselmis* sp. or *Spirulina* sp. either live, frozen or dried [18–21]. This is sometimes supplemented in later stages by a protein source such as *Artemia* sp. brine shrimp or rotifers and shredded spinach leaves, which represent a macroalgae substitute. *Platynereis dumerilii* are usually fed from approximately one week of age [19,20]. This led us to question whether a potential food source may act as a natural settlement cue for *P. dumerilii* larvae? We hypothesized that if *P. dumerilii* would encounter such a cue earlier on in their development, even before they had developed the capacity to feed, they could transition out of their planktonic phase sooner, through a larval settlement process.

Previous studies of the diet of *P. dumerilii* based on a natural population of worms from the Gulf of Naples (where laboratory cultures were originally sourced [5]) found that small worms primarily eat epiphytic microalgae growing on the macroalgal beds they inhabited, including diatoms and coccolithophores [22,23]. Microbial marine biofilms are dominated by bacteria and diatoms (unicellular microalgae of the group Bacillariophyceae) [24]. Microbial biofilms are a common inducer of larval settlement across many marine invertebrate phyla, including annelids [25,26]. Studies of larval settlement and metamorphosis in the polychaete *Hydroides elegans* previously identified both diatoms and bacteria as inductive cues for larval settlement [27–29]. More recent studies have particularly emphasized a key role for bacteria as inducers of settlement and metamorphosis, through a variety of species-specific mechanisms, including a contractile injection system [30–32], and lipopolysaccharide-containing vesicles [33]. These findings, together with the known dietary preferences of *P. dumerilii*, suggest that microbial biofilms may also play an inductive role in *P. dumerilii* larval settlement.

The study and targeted use of microbial biofilms as a cue for larval settlement and metamorphosis can aid in improving production within invertebrate aquaculture settings. For example, the addition of diatoms to settlement plates for the abalone, *Haliotis iris*, effectively doubled the percentage of larval settlement compared with control plates lacking diatoms [34]. Pre-conditioning collectors for larval scallops with a specific combination of *Halomonas* sp. bacterium and *Amphora* sp. diatoms promoted settlement of the larval scallops better than naturally deposited microbial films [35]. A benthic diatom, *Amphora helenensis*, was identified as enhancing larval settlement, metamorphosis and early growth of juvenile sea urchins (*Lytechinus variegatus*) due to its nutritional role [36]. The importance of microalgae in aquaculture cannot be understated; the productivity of a larval hatchery is directly related to the quality of microalgae employed as both a food source and a settlement cue [37].

Here, we investigate microbial biofilms as potential larval settlement cues for *P. dumerilii* larvae through a series of settlement assays. We base initial tests on biofilms developed from our adult culture boxes of *P. dumerilii*, then address individual components of biofilm, including bacteria, diatoms, green microalgae and coccolithophores, to determine from which source in the biofilm inductive cues may arise. Having established an important role for microalgae, particularly diatoms, in the induction of *P. dumerilii* settlement, we then ask whether this effect is species specific. Finally, given the role of microalgae in larval settlement and its link to the early juvenile diet, we ask whether the inclusion of diatoms in the early diet of *P. dumerilii* laboratory cultures can enhance growth in their first month of culture.

## 2. Material and methods

### 2.1. *Platynereis dumerilii* culture

*Platynereis dumerilii* were cultured in the Marine Invertebrate Culture Unit (MICU) at the University of Exeter using a culturing method adapted from that described by Fischer & Dorresteijn [19]. Detailed information regarding *P. dumerilii* culture is provided in the electronic supplementary material, Methods; supplementary data.

**Figure 1.**
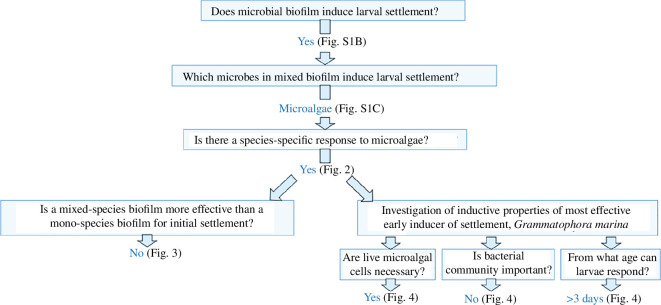
Overview of *P. dumerilii* larval settlement assays and the questions addressed in this study.

Larvae used for the settlement assays were raised in 100 ml glass beakers in an incubator on a 16 : 8 h light/dark cycle at 18℃. A schematic overview of our experimental questions and progression can be seen in figure 1. Methods for initial investigation of mixed species microbial biofilms sourced from adult *P. dumerilii* culture boxes are included in electronic supplementary material, Methods; supplementary data.

### 2.2. Microalgae culture

Diatoms and other microalgae species were cultured in 50 ml vented flasks (BioLite 25 cm^2^, ThermoFisher Scientific 130 189) with 40 ml culture medium (autoclaved 0.22 micron-filtered and UV-sterilised artificial sea water (ASW, Tropic Marin Pro-Reef salt) containing F/2 nutrient medium at 1 ml per 2.5 l (ZM Systems), and silicate solution at 1 ml per 2.5 l (ZM Systems) at 18℃ in an incubator with fluorescent overhead illumination under a 16 : 8 h light : dark cycle.

Each strain was subcultured every three weeks by transferring 5–10 ml culture into a new flask and topping up to 40 ml with fresh culture medium. All work with microalgae cultures was carried out in a microbiological cabinet to prevent cross-contamination of cultures.

For single-species microalgae biofilm assays, algae stock cultures were obtained through collaborators, purchase or through an ASSEMBLE Plus Remote Access Grant from microalgal culture collections in the UK and Europe (Marine Biological Association (MBA)) UK; Culture Collection of Algae and Protozoan (CCAP), Scotland; Roscoff Culture Collection (RCC), France; Stazione Zoologica Napoli (SZN), Italy and the Belgian Coordinated Collection of Microorganisms (BCCM/DCG), Ghent. Further information on microalgal IDs and characteristics is provided in electronic supplementary material, table S4. Note that microalgal cultures, although consisting predominantly of a single species, were not guaranteed to be axenic, and may have contained other microorganisms such as bacteria and ciliates, if these were present in the original stock cultures provided.

### 2.3. Preparation of microalgal biofilms for settlement assays

Plastic coverslips were prepared from A4 sheets of Aclar film (Agar Scientific AGL4458) with a 12.7 mm disc punch (Agar Scientific AGT5443). Discs were autoclaved to ensure sterility and subsequently coated with poly-D-lysine to improve adherence of the biofilm cells. For poly-D-lysine coating, coverslips were submerged in a solution of 10 µg ml^−1^ poly-D-lysine (Sigma-Aldrich P6407) at room temperature for 1 h, rinsed three times briefly in MilliQ H_2_O, then air-dried and stored at 4℃ for up to one month before use in settlement assays.

To generate microalgae biofilms, cell suspensions of each microalgae strain were aliquoted into 24-well plates (ThermoFisher Scientific 930 186 BioLite 6 well multidish) containing a round coverslip at 1.5 ml/well and incubated overnight at 18°C. Unattached microalgae cells were removed by gently dip-rinsing coverslips three times in 50 ml sterile seawater. Negative control slips were also generated by incubating coverslips overnight with 1.5 ml diatom culture medium (artificial seawater with F/2 and silica) and dip-rinsing three times before transfer to 6-well plates for settlement assays. For tests of the effect of *Grammatophora marina* biofilm age, six coverslips were additionally incubated following dip-rinsing for one week, two weeks and one month in the 24-well plates, with twice weekly change of culture medium to allow further development of the biofilm. We did not adjust microalgae culture cell density before addition to coverslips as cell density in the biofilm was expected to vary due to differences in the adherence and physical properties (e.g. size and shape) of different microalgae species. For each species, we calculated representative cell densities in the biofilm by imaging a 2.6 mm area for each of three coverslips per strain using a Zeiss Axiozoom v. 18 (Carl Zeiss GmbH) with a digital CMOS camera ORCA-Flash-4.0 (Hamamatsu) and 2.3× objective at 100× magnification. Example images of biofilms can be seen in electronic supplementary material, figures S4 and S8. Mean densities for each strain’s biofilm were calculated from these images using a custom ImageJ macro (available at https://github.com/MolMarSys-Lab/Platynereis-diatom_Hird_et_al_2024/tree/main/macros) to calculate the percentage area of cell coverage.

### 2.4. Larval settlement assays

Larval settlement assays were carried out in 6-well plates (ThermoFisher Scientific 130 184 BioLite 6-well multidish) with 10 ml FSW and 30 *P. dumerilii* nectochaete larvae per well. Larvae were sourced from a mixture of three different larval batches generated by crossing individual male and female *P. dumerilii* epitokes. Larvae were introduced to the 6-well plates containing biofilmed or negative control coverslips at the start of each experiment by pipetting with a P10 micropipette (Gilson). To investigate the species-specific settlement response of *P. dumerilii* to monospecies microalgal biofilms, with a particular focus on diatom species, three replicates of 33-day-old nectochaete larvae were tested on three independent occasions, with 9 × 30 larvae tested overall per species. Numbers of larval settlement, death and swimming larvae were scored as above at 24 and 48 h after the addition of larvae to the experimental plates.

For further investigations of the effects of mixed species biofilms and further characterization of the settlement response induced by the most effective diatom species, *G. marina*, additional settlement and growth assays were carried out. To test whether a mixed species biofilm could further increase settlement rate through synergistic effects, we carried out settlement assays comparing percentage larval settlement of 3-day-old *P. dumerilii* exposed to either single-species biofilms of *G. marina* and *Achnanthes brevipes* or to various combinations of multiple species of microalgae. We tested four different microalgae mixtures: (i) a ‘settlement formula’ consisting of three species that induced high levels of settlement after 24 h as single-species biofilms (*G. marina*, *A. brevipes*, *Nitzschia ovalis*), (ii) another ‘settlement formula’ consisting of six species that induced significant levels of settlement after 24 h as single-species biofilms (*G. marina*, *A. brevipes*, *N. ovalis, Achnanthes* sp*., Chrysotila lamellosa, Fragilaria striatula*), (iii) a ‘growth formula’ consisting of six species that induced highest levels of growth after 11 days (*G. marina, N, ovalis, A. brevipes, Nitzschia laevis, Amphora* sp. (Roscoff Culture Collection RCC70623), *Achnanthes* sp.), and (iv) another ‘growth formula’ consisting of 10 species that induced significant growth after 11 days (*G. marina, N, ovalis, A. brevipes, N. laevis, Amphora* sp. (Roscoff Culture Collection RCC70623), *Achnanthes* sp., *C. lamellosa, F. striatula, Skeletonema dohrnii, Gomphonema* sp.). Biofilmed coverslips were prepared as above and allowed to develop overnight. For mixed biofilms, equal volumes of each individual species were pre-mixed in 15 ml falcon tubes before transfer to 24-well plates containing round coverslips. Numbers of larval settlement, death and swimming larvae were scored as above at 24 and 48 h after the addition of larvae to the experimental 6-well plates. Six replicates of 30 larvae were tested and scored at 24 and 48 h. Alongside this, to determine the impact of biofilm age on larval settlement, we tested 3-day-old *P. dumerilii* larval settlement response to single-species *G. marina* biofilms of different ages, with three replicates of 30 larvae scored at 24 and 48 h.

Further investigation of the inductive properties of the 1-day-old *G. marina* biofilm was also carried out with six replicates of 30 larvae per control and treatment, and scoring of larval responses as above. To test the inductive properties of the 1-day-old *G. marina* biofilm, and the relative contribution of viable diatom cells and other microbial cells, including bacteria, in the biofilm, we subjected *G. marina* biofilmed coverslips to the following treatments before use in settlement assays, with dip-rinsing before and after treatments: (i) 1 µm filtration of microalgae cell suspension (Millipore 1 µM filter SLFAO5010), to remove diatom cells but retain microbial cells, (ii) submerged in 100% EtOH for 30 min, (iii) submerged in 100℃ seawater for 30 min, (iv) submerged in 50℃ seawater overnight, (v) dried at 50℃ overnight, (vi) treated with antibiotics (penicillin-streptomycin, Sigma P4333, 5 units penicillin, 5 µg ml^−1^ streptomycin) at the starting point of settlement assay, and (vii) treated with antibiotics both prior to biofilm formation (10 units penicillin, 10 µg ml^−1^ streptomycin) and at starting point of settlement assay (5 units penicillin, 5 µg ml^−1^ streptomycin). Antibiotic treatments were intended to alter the composition of the bacterial communities associated with the diatom biofilm and provide information on the role of the bacterial community in *P. dumerilii* larval settlement. Treatments were based on similar experiments performed by Bao *et al*. [38] to investigate the settlement response of mussel larvae to biofilm cue. To calculate viable cell densities in the treated biofilms, we imaged them on an Axiozoom v. 18 stereo microscope as described above, recording images under both white light and fluorescent light emission at 535 nm. Example images of biofilms can be seen in electronic supplementary material, figures S4 and S12.

To investigate the influence of larval age on the settlement response induced by the 1-day-old *G. marina* biofilm, we also performed settlement assays with larvae introduced to a well with a biofilmed coverslip at 2, 3, 3.5, 4, 5, 6, 7 or 8 days old. Six replicates of 30 larvae were tested and scored at 24 and 48 h after induction for each age group. As previous *G. marina* treatment assays indicated a positive effect of antibiotic treatment on larval survival and settlement, we incorporated antibiotic treatment into these larval age assays, with penicillin-streptomycin (Sigma P4333, 5 units penicillin, 5 µg ml^−1^ streptomycin) added to the wells with larvae at the time of induction.

### 2.5. Larval growth measurements

In addition to scoring larval settlement in assays at 24 and 48 h after induction, we measured the size of juveniles from settlement assays at 8 days after induction to investigate the effect of settlement biofilm on growth. For this, we maintained the larvae from settlement assays in their 6-well plates for 8 days after induction, exchanging 3 ml artificial seawater (ASW) every 2 days with a micropipette. We measured the body length and segment number of representative individuals by imaging individuals relaxed by drop-wise application of 1 M MgCl_2_ in ASW using a Zeiss Stemi 508 stereo microscope at 3× magnification with Zeiss Axiocam 208 colour camera and Zeiss Labscope software. Measurements of images were taken manually in ImageJ v. 1.54 f using the line tool and ‘measure’ analysis. For each well of a 6-well settlement assay plate that initially contained 30 larvae, six representative 11-day-old individuals were selected for measurement, based on their proximity to the centre of the coverslip in the well at the time of imaging. Size measurements were recorded for larvae from the testing of 40 different microalgae single-species biofilms, and the testing of mixed versus single-species biofilms. Example images of *P*. *dumerilii* at 11 days old can be seen in electronic supplementary material, figures S5 and S9.

### 2.6. Use of diatoms in large-scale culture of *Platynereis*

Given the positive effect of several microalgae species on the initial growth of *P. dumerilii* larvae, we decided to test whether incorporating one or two diatom species into our regular culture of *P. dumerilii* could also enhance longer term growth on this larger scale. We focused on effects in the first 30 days of culture, as during this time period the worms are fed microalgae only, whereas from 30 days, the worms receive additional nutrition in the form of rotifers and spinach. Our aim in this case was not to test the effects of the diatom species most effective for early settlement but to establish whether the addition of diatoms in general to the *P. dumerilii* diet could enhance longer term growth. Diatom species chosen for this study were *Phaeodactylum tricornutum* (PLY100) and *Skeletonema dohrnii* (PLY612) due to their ability to be cultured easily on a large scale in our microalgal aquaculture set-up and their common and widespread use as food in several aquaculture environments [36]. These were tested alongside common large-scale aquaculture strains of microalgae already established for marine invertebrate nutrition, including *Tetraselmis suecica* (CCAP66/4), *Nannochloropsis salina* (CCAP849/3) and *Isochrysis galbana* (CCAP927/20). Detailed information on large-scale culture of marine microalgae is provided in electronic supplementary material, Methods; supplementary data.

Four microalgal feed treatments were established for testing: (i) a single non-diatom species (*Tetraselmis suecica* or *Nannochloropsis salina*); (ii) a single diatom species (*Phaedoactylum tricornutum* or *Skeletonema dohrnii*); (iii) mixed species microalgal feed consisting of three species (*T. suecica, N. salina* and *P. tricornutum*) and (iv) mixed species microalgal feed consisting of five species (*T. suecica, N. salina, Isochrysis galbana, P. tricornutum* and *S. dohrnii*). For the single-species treatments, worms were fed on one or the other of the two listed species, depending on which was in the culture at the point of establishing that replicate. Mixed species treatments consisted of equal volumes of each component species.

Worms for the culturing trials were maintained unfed in an incubator at 18°C until they reached 7 days old, at which point they were cultured in 4 l styrene-acrylonitrile (SAN) boxes containing 1.5 l ASW at a stocking density of 80 worms per box, established by individually pipetting worms over. Each box was assigned to one of the four aforementioned feeding regimes and fed by the addition of 10 ml of the relevant microalgal mix, three times per week, commencing from 7 days post-fertilization.

At 30 days old, a maximum of 10 worms per culture box were removed, immobilized by transferring them into a 1 M MgCl_2_ in ASW solution, and the number of segments of each worm was counted under a Zeiss Stemi 508 stereo microscope and the 0–30 day growth rate established by dividing the total number of segments by 30 days.

### 2.7. Statistical analyses

Data analysis and plotting were carried out in Python 3, using pandas and seaborn packages [39,40]. Scripts and raw data files for data analysis and plots are available on Github at https://github.com/MolMarSys-Lab/Platynereis-diatom_Hird_et_al_2024.

**Figure 2.**
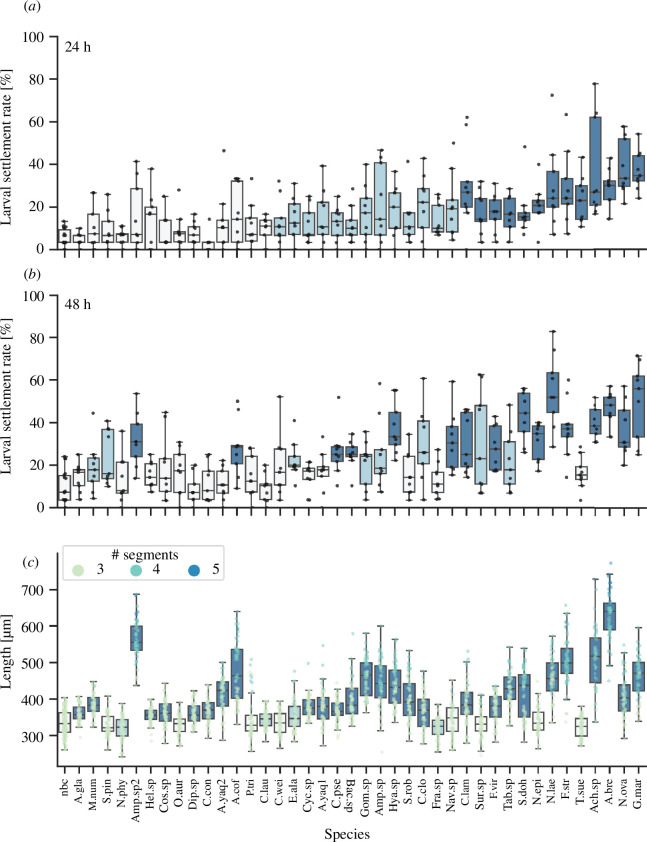
*Platynereis dumerilii* show a species-specific response to microalgae biofilms as inductive cues for settlement. Box plots with scatter plot overlay of (*a*) percentage larval settlement in response to different monospecies microalgal biofilms after 24 h and (*b*) 48 h exposure and (*c*) the total length of individual larvae exposed to the same biofilms for 8 days. Scatterplot points in (*c*) are coloured by the segment number of each larva. Boxplots are coloured according to the *p*-value result of a Mann–Whitney U rank test of percentage larval settlement at 24 and 48 h, or total length at 8 days, in each species versus the no biofilm control; *p* > 0.05 = pale grey, *p* < 0.05>0.00125 = light blue, *p* < 0.00125 = dark blue. A.gla = *Asterionellopsis* cf. *glacialis*, M.num = *Melosira nummuloides*, S.pin = *Staurosirella pinnata*, N.phy = *Navicula phyllepta*, Amp.sp2 = *Amphora* sp. RCC7063, Hel.sp = *Helicotheca* sp., Cos.sp = *Coscinodiscus* sp., O.aur = *Odontella aurita*, Dip.sp = *Diploneis* sp., C.con = *Chaetoceros convolutus*, A.yaq2 = *Achnanthes yaquinensis* DCG0053, A.cof = *Amphora coffeaeformis*, P.tri = *Phaeodactylum tricornutum*, C.lau = *Chaetoceros* cf. *lauderi*, C.wei = *Conticribra weisflogii*, E.ala = *Entomoneis alata*, Cyc.sp = *Cyclotella* sp., A.yaq1 = *Achnanthes yaquinensis* DCG0020, C.pse = *Chaetoceros pseudocurvisetus*, Bac.sp = *Bacteriastrum* sp., Gom.sp = *Gomphonema* sp., Amp.sp = *Amphora* sp., Hya.sp = *Hyalosira* sp., S.rob = *Seminavis robusta*, C.clo = *Cylindrotheca closterium*, Fra.sp = *Fragilariopsis* sp., Nav.sp = *Navicula* sp., C.lam = *Chrysotila lamellosa*, Sur.sp = *Surirella* sp., F.vir = *Fragilariformia virescens*, Tab.sp = *Tabularia* sp., S.doh = *Skeletonema dohrnii*, N.epi = *Nitzschia epithemoides*, N.lae = *Nitzschia laevis*, F.str = *Fragilaria striatula*, T.sue = *Tetraselmis suecica*, Ach.sp = *Achnanthes* sp., A.bre = *Achnanthes brevipes*, N.ova = *Nitzschia ovalis*, G.mar = *Grammatophora marina*, nbc = no biofilm control.

Each dataset was initially checked for normality using a Shapiro–Wilk test, which confirmed non-normal distributions. A Kruskal–Wallis test was used to assess significant differences in treatment and control in initial mixed biofilm experiments. For subsequent species-specific settlement assays, a Mann–Whitney U rank test was used to test for significant differences in both percentage larval settlement between each species and the negative control. A Bonferroni correction was applied to the *p*-value cut-off when comparing larval settlement across 40 different microalgae species (figure 2), to account for potential false positives with this higher sample number. The correlation between percentage larval settlement and biofilm percentage coverage was tested using Kendall’s tau test, both for all 40 microalgae species tested, and for the subset of microalgae found to be significantly inductive at at least one time point according to the Mann–Whitney U rank test with Bonferroni correction. We also applied the Mann–Whitney U rank test with Bonferroni correction to investigate whether the percentage of larvae dead at 24 and 48 h was significantly different in any microalgae species compared with the negative control.

A Kruskal–Wallis test with Dunn’s post hoc test was used to investigate whether the initial settlement responses of *P. dumerilii* at 24 h varied according to specific features of microalgae including group, cell shape, cell assemblage and cell size. To assess whether the encounter with the 40 species of algae led to significantly different growth by 11 days of age compared with the no biofilm control samples, we applied a Mann–Whitney U rank test with Bonferroni correction. Subsequent experiments were also analysed for significant differences to the control using the Mann–Whitney U rank test, and the Kruskal–Wallis test with Dunn’s post hoc test was used to check for significant differences between all potential pairwise comparisons. For each experiment, we recorded biofilm densities as described above and used these to check for any correlation with larval settlement rate using Kendall’s tau test.

## 3. Results

Initial testing of the settlement rate of *P. dumerilii* larvae in response to mixed microbial biofilms grown in adult worm culture boxes indicated that larvae settle earlier in the presence of a mixed microbial biofilm than they would in the absence of this environmental cue (electronic supplementary material, figure S1b). Subsequent testing of the effects of individual bacterial species isolated from the biofilm, and individual species of microalgae including green microalgae (*Tetraselmis suecica*), coccolithophores (*Chrysotila lamellosa*) and diatoms (*Phaeodactylum tricornutum*, *Skeletonema dohrnii* and *Amphora coffeaeformis*) showed that bacterial biofilms alone did not induce significant levels of *P. dumerilii* settlement, but biofilms of individual microalgae species did induce significantly higher rates of settlement compared with the control coverslip that lacked a biofilm (electronic supplementary material, figure S1c). Unlike the green microalgae *T. suecica*, which induced relatively low levels of settlement, single-species diatom and coccolithophore biofilms were capable of inducing equal or greater numbers of larval settlement as the original mixed biofilm sourced from the adult worm culture boxes.

### 3.1. Single-species microalgal biofilm

Having established that the primary inductive component in a mixed microbial biofilm was probably of microalgal origin, we then proceeded to test a number of additional different single-species biofilms in settlement assays, with additional replication across multiple batches of *P. dumerilii* larvae, to determine whether the settlement response of *P. dumerilii* to microalgae was species specific. Of the 40 species tested, we found that the species that induced the greatest median settlement after both 24 and 48 h was the chain-forming diatom *G. marina*. According to a Mann–Whitney U rank test with Bonferroni correction, of the 40 microalgae species tested, 13 species induced significantly greater larval settlement after 24 h compared with a no biofilm control, with 10 of these 13 species maintaining significantly greater levels of larval settlement 48 h into the assay (figure 2*a*,*b*). Apart from *G. marina*, other highly inductive microalgae species were *Nitzschia* species, including *N. ovalis*, *N. laevis* and *N. epithemoides*, and *Achnanthes* species, including *Achnanthes* sp. and *A. brevipes*. Larval settlement response was also species specific in that different microalgae species from the same genus elicited significantly different levels of *P. dumerilii* larval settlement. For example, two strains of *Achnanthes yaquinensis* tested did not induce significant larval settlement at 24 and 48 h, while *Achnanthes* sp. and *A. brevipes* did.

Based on the results of Kruskal–Wallis tests with Dunn’s post hoc testing, the initial settlement responses of *Platynereis* larvae to different microalgal species varied significantly depending on the cell shape, size and phylogenetic group of the species tested (electronic supplementary material, figure S4). Round or centric diatom species induced significantly lower levels of larval settlement overall. The results of these tests indicate that the settlement response of *Platynereis* larvae is most strongly induced by pennate chain-forming diatoms under 50 μm in length. According to a Mann–Whitney U rank test with Bonferroni correction, no microalgae strains caused significantly more larval death than the control scenario (electronic supplementary material, figure S1 and table S6). Comparing percentage larval settlement with the percentage biofilm coverage of each microalgae species tested using Kendall’s rank correlation, we found a significant but negligible positive correlation between biofilm coverage and the rate of larval settlement (electronic supplementary material, figure S3), leading us to conclude that biofilm coverage is not of primary importance to predicting settlement rate.

**Figure 3.**
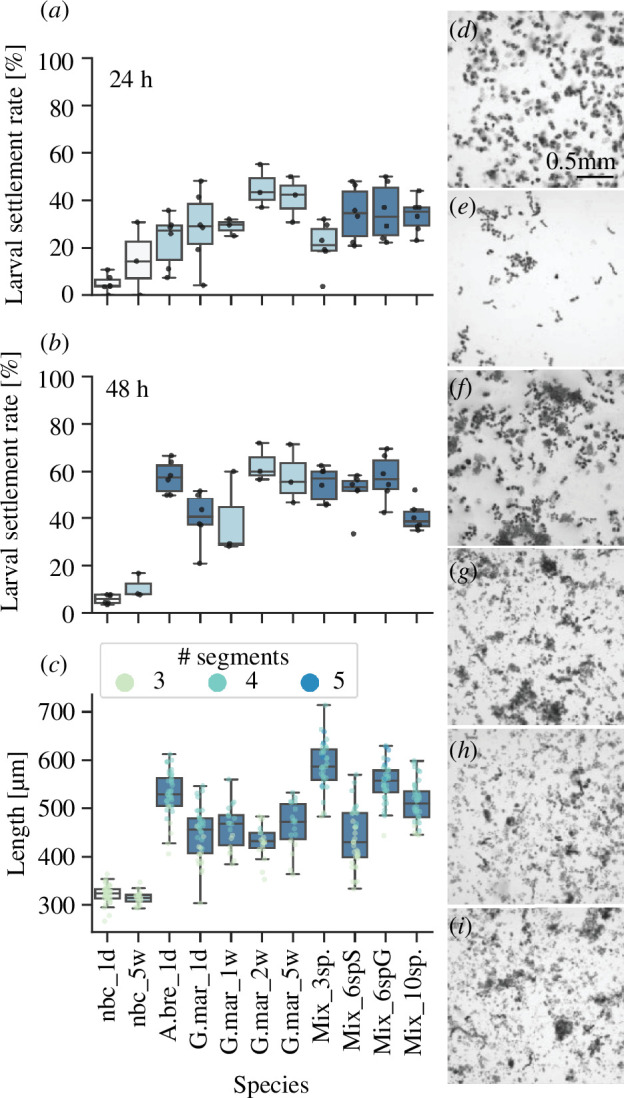
Impact of biofilm age and composition on *Platynereis dumerilii* larval settlement and early growth. Box plots with scatter plot overlay of (*a*) percentage larval settlement in response to different monospecies or mixed microalgal biofilms after 24 h and (*b*) 48 h exposure and (*c*) the total length of individual larvae exposed to the same biofilms for 8 days. Scatterplot points in (*c*) are coloured by the segment number of each larva. Boxplots are coloured according to the *p*-value result of a Mann–Whitney U rank test of percentage larval settlement at 24 and 48 h, or total length at 8 days, in each species versus the no biofilm control; *p* > 0.05 = pale grey, 0.005 < p < 0.05 = light blue, *p* < 0.005 = dark blue. (*d*–*i*) Light micrograph example images of different monospecies and mixed biofilms used in settlement assays. (*d*) *Achnanthes brevipes*, (*e*) *Grammatophora marina*, (*f*) three species mixed biofilm, (*g*) six species mixed biofilm, targeted for induction of early settlement, (*h*) six species mixed biofilm, targeted for induction of both early settlement and longer term growth, (*i*) 10 species mixed biofilm. A.bre_1d = *A. brevipes* 1-day-old biofilm, G.mar_1d = *G. marina* 1-day-old biofilm, G.mar_1w = *G. marina* 1-week-old biofilm, G.mar_2w = *G. marina* two-week-old biofilm, G.mar_5w = *G. marina* five-week-old biofilm, Mix_3sp. = mix of three diatom species, Mix_6spS = mix of six microalgae species selected for positive effects on early settlement, Mix_6spG = mix of six microalgae species with positive effects on growth by 11 days, Mix_10sp. = mix of 10 microalgae species, nbc_1d = no biofilm control 1 day old, nbc_5w = no biofilm control five weeks old.

Although *G. marina* induced the highest median settlement rates in *P. dumerilii* larvae at 24 and 48 h after induction, biofilms of this species did not support the highest median growth over the week following settlement. Of the 40 strains tested, 28 induced significantly more growth by 11 days compared with a no biofilm control (figure 2*c*). Top inducers of initial settlement, *G. marina*, *N. ovalis* and *A. brevipes* all induced significant growth after 11 days compared with control. Although not an inducer of significantly higher early settlement, *Amphora* sp. RCC7063 did induce significant growth by 11 days, and was one of the few species in which we found juveniles with five segments present. Other species that enabled the growth of juvenile *Platynereis* to five segments by 11 days were *Amphora coffeaeformis*, *Fragilaria striatula*, *Achnanthes brevipes* and *Achnanthes* sp.

### 3.2. Biofilm age and composition

In subsequent settlement and growth assays, we investigated the effects of biofilm age and composition on larval settlement rates in *P. dumerilii*. Here, we focused primarily on *G. marina* biofilms, as this species induced the highest median percentage larval settlement in previous assays. Overall, the age of *G. marina* biofilm did not significantly alter the settlement rates of *P. dumerilii* larvae over 48 h, or the growth achieved by 11 days (figure 3; electronic supplementary material, figure S8 and tables S9 and S10). Similarly, although mixed species biofilms induced significantly greater larval settlement at 24 h compared with the no biofilm control, they did not cause significantly greater larval settlement at 24 or 48 h compared with single-species biofilms of either *G. marina* or *A. brevipes*. However, mixtures of three diatom species selected based on their positive effects on larval settlement in initial testing, or six diatom species selected for their positive effects on larval growth in initial testing, did induce significantly more growth in larvae by 11 days compared with larvae exposed to either a single-species *G. marina* biofilm, a mixed biofilm of six species selected for positive effects on settlement or a mixed biofilm of 10 species selected for their positive effects on settlement and growth. Growth by 11 days in larvae exposed to the single species *A. brevipes* biofilm was equivalent to that shown by larvae in the best mixed species biofilms. As with the initial single-species testing, biofilms tested in the biofilm age and composition settlement assays did not cause significantly greater rates of larval death (electronic supplementary material, figure S7 and table S9), and there was no significant correlation between the density of biofilms tested and larval settlement rates (electronic supplementary material, figure S8).

### 3.3. *Grammatophora marina* biofilm

**Figure 4.**
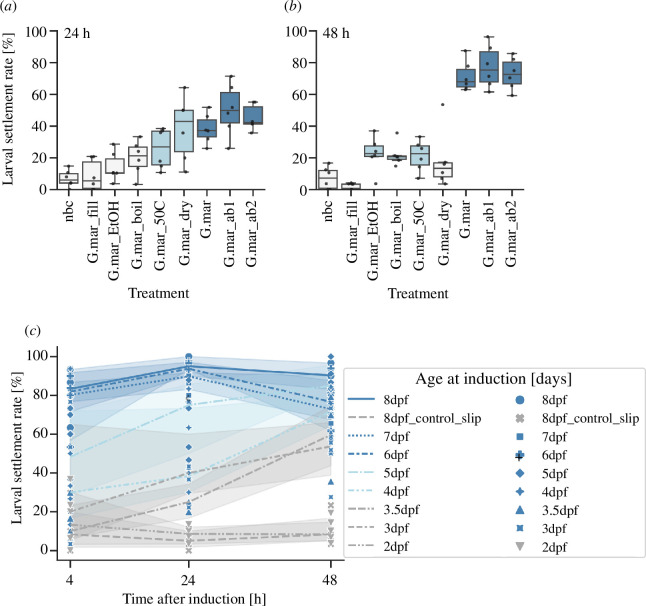
Characterization of *P. dumerilii* larval settlement in response to *G. marina* biofilm. Box plots with scatter plot overlay of (*a*) percentage larval settlement in response to *G. marina* monospecies biofilms subjected to different treatments after 24 h and (*b*) 48 h exposure. Boxplots coloured according to the *p*-value result of a Mann–Whitney U rank test of percentage larval settlement at 24 and 48 h, in each treatment versus the no biofilm control; *p* > 0.05 = pale grey, 0.00625 < *p* < 0.05 = light blue, *p* < 0.00625 = dark blue. G.mar_fil = *G. marina* filtrate, G.mar_EtOH = EtOH-treated *G. marina*, G.mar_boil = boiled *G. marina*, G.mar_dry = *G. marina* 50℃ overnight (dry), G.mar_50C = *G. marina* 50℃ overnight (submerged), G.mar = 1-day-old untreated *G. marina* biofilm, G.mar_ab1 = *G. marina* treated with antibiotics after biofilm formation, G.mar_ab2 = *G. marina* treated with antibiotics before and after biofilm formation, nbc = no biofilm control. (*c*) A line plot with scatter plot overlay of median percentage larval settlement over time for larvae introduced to G. marina biofilm at different initial ages (2–8 days old). Line shadows indicate 95% confidence interval. Lines are coloured according to the *p*-value result of a Mann–Whitney U rank test of percentage larval settlement at 4 h in each age group versus 8 days old larvae with a no biofilm control; *p* > 0.05 = grey, 0.00625 < *p* < 0.05 = light blue, *p* < 0.00625 = dark blue. Raw data points coloured according to the *p*-value result of a Mann–Whitney U rank test of percentage larval settlement at 24 h in each age group versus 8 days old larvae with a no biofilm control; *p* > 0.05 = grey, 0.00625 < *p* < 0.05 = light blue, *p* < 0.00625 = dark blue, (dpf = days post-fertilization).

Having established that mixed microalgae biofilms do not have a synergistic effect on *P. dumerilii* larval settlement compared with the most inductive single-species biofilms, we next carried out settlement assays to further characterize the inductive properties of the most effective species of diatom biofilm, *G. marina*. We tested *G. marina* biofilms treated with heat or ethanol to reduce cell viability. We also tested *G. marina* filtrate and *G. marina* biofilm treated with antibiotics to separate any bacterial components from the diatom cell components. The antibiotic treatment of *G. marina* biofilm either before and after the biofilm formation, or only after biofilm formation did not significantly alter the inductive capacity of the biofilm, while the filtrate of *G. marina*, which contained bacterial, but not diatom cells, did not induce significantly higher levels of *P. dumerilii* settlement than the no biofilm control (figure 4*a*,b). *Grammatophora marina* biofilms with reduced cell viability showed reduced levels of *P. dumerilii* settlement at 24 and 48 h after induction. In particular, the biofilm treated with EtOH or heated to 100℃ induced levels of settlement similar to the no biofilm control. In these settlement assays, *G. marina* biofilm density, both of total cells and viable cells, showed a significant strong positive correlation with larval settlement rate according to Kendall’s tau test (electronic supplementary material, figure S12). Ethanol, heat and antibiotic treatment of the *G. marina* biofilm all resulted in a small but significant reduction in death rate across the 48 h of the assays (electronic supplementary material, figure S11 and table S12).

Settlement assays exposing *P. dumerilii* larvae of different ages to 1-day-old *G. marina* biofilm indicated that the capacity for larvae to respond to microalgal biofilms increases with age (figure 3*c*). Worms between 4 and 8 days of age show significant increases in larval settlement rate compared with the no biofilm control even just 4 h after the start of the assay. In 6–8-day-old worms, between approximately 60 and 90% of worms had already settled on the *G. marina* biofilm within 4 h. In worms 3 days and older, significant levels of settlement were seen from 24 h after the start of the assay, but worms introduced to the assay at 2 days old never showed significantly increased rates of larval settlement across the 48 h of the assay. In the absence of a diatom biofilm, worms that were 8 days old at the start of the assay showed up to approximately 20% settlement after 48 h, whereas 8-day-old worms in the presence of *G. marina* biofilm reached a maximum of approximately 96% settlement after 48 h.

### 3.4. Diatom effects on *Platynereis* growth

**Figure 5.**
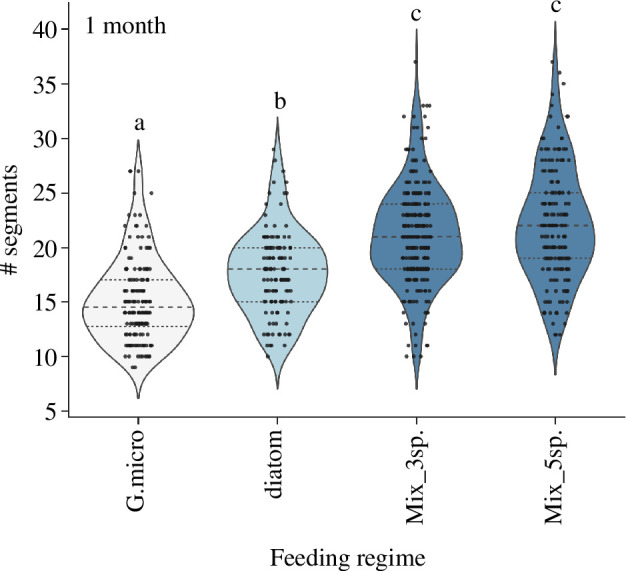
Effects of incorporation of diatoms into large-scale *Platynereis dumerilii* culture. Violin plots with strip plot overlay of number of segments in individual 30-day-old worms raised from 7 days on a single-species green microalga (G.micro), a single-species diatom (diatom), a mixed culture of two microalgae and one diatom species (Mix_3sp.) or a mixed culture of three microalgae and two diatoms (Mix_5sp.). Different feeding regimes are coloured based on their significance groups according to Kruskal–Wallis with Dunn’s post hoc testing, which is also indicated above each group with ‘a’, ‘b’ or ‘c’. G.micro = green microalgae, Mix_3sp. = two microalgae plus one diatom species, Mix_5sp. = three microalgae plus two diatom species.

Similar to the positive effect of diatom species on early larval settlement, providing a diatom food source for *P. dumerilii* following settlement also boosted growth of the worms over the first month of their life in our large-scale culture (figure 5). Feeding young worms between one week and one month with a single diatom species (either *Phaeodactylum tricornutum* or *Skeletonema dohrnii*) increased size at 30 days by an additional approximately two segments on average compared with feeding with a single green microalgal species (*Tetraselmis suecica* or *Nannochloropsis salina*). Highest growth after 30 days was seen in worms fed a mixed diet of either three microalgal species including one diatom species, or of five microalgal species including two diatom species; however, increasing the species diversity in the mixed diet from three to five did not significantly change worm size. The median size achieved on mixed diets was 22–23 segments by 30 days. The largest worms recorded in these feeding trials had 37 segments. Worms of this size occurred under both mixed feeding regimes. The overall variance in size at 30 days under all diets was quite high, ranging from approximately 14 segments in worms fed single-species diets, to approximately 26 segments in worms fed a mixed diet of five different microalgae species.

## 4. Discussion

In this study, we investigated a potential role for microbial biofilm in the induction of larval settlement of the marine worm *P. dumerilii*. Through a series of behavioural assays, we established that microbial biofilm can induce an earlier onset of settlement and transition to a benthic lifestyle in the developing larva. The main component of biofilm that attracts larvae is microalgae, particularly diatoms, and *P. dumerilii* larvae respond to these microalgae in a species-specific manner.

### 4.1. Microalgae as a larval settlement cue

Diatoms are already known to induce larval settlement in a species-specific manner in several other marine invertebrate larvae, although identification of species preferences is always limited by the number of species tested in each study. For example, mixed or monospecies diatom biofilms induced settlement and subsequent metamorphosis in the clam *Macoma balthica* [41], the polychaete *Hydroides elegans* [27,28,42], the bryozoan *Bugula neritina* [43,44], the slipper limpet *Crepidula onyx* [45], various abalone *Haliotis* spp. [46–49], the scallop *Argopecten purpuratus* [50], the acorn barnacle *Balanus amphitrite* [51,52] and the gastropod *Ilyanassa obsoleta* [53]. Due to their positive effect on settlement, metamorphosis and subsequent juvenile growth, settlement plates coated with diatom-dominated biofilm are often implemented in shellfish and echinoderm aquaculture to enhance recruitment of spat [48,54–56]. A growing body of evidence, including the results of this study, suggests that juvenile recruitment may be further optimized by selectively matching invertebrate larvae with their unique combination of preferred microalgal species.

As seems to be the case for other marine invertebrates whose larval settlement is induced by diatoms, *P. dumerilii* larvae were particularly attracted to settle on biofilms dominated by benthic pennate diatom species including *Achnanthes* and *Nitzschia* species. Microalgae that induced higher levels of *P. dumerilii* settlement were not necessarily closely related to each other, but the best inducers, including the coccolithophore *Chrysotila lamellosa*, share a common feature of secreting polysaccharide-rich extracellular polymeric substances (EPS) [57–59], suggesting that the EPS may be a significant element within the biofilm that attracts *P. dumerilii*. The EPS was previously identified as being responsible for the inductive effect of diatom biofilms on larval settlement in *Hydroides elegans* [42], *Macoma balthica* [41] and *Balanus amphitrite* [51]. Our settlement assays with *G. marina* biofilm treated with heat or ethanol to reduce viability indicate that a metabolically active biofilm is required to maintain inductive cue levels at a concentration high enough to be detected by the *P. dumerilii* larvae. A similar system has been proposed for the induction of *Hydroides elegans* larval settlement by bacterial biofilms [60].

We consistently recorded the highest levels of settlement in response to the chain-forming diatom species *G. marina*, although rates of larval settlement in response to this species were only marginally higher than those induced by *Achnanthes* and *Nitzschia* species. The fact that *G. marina* biofilm was equally effective in inducing initial *P. dumerilii* larval settlement as various mixed species biofilms and that the effect was not significantly impacted by biofilm cell density or age suggests that this species may produce a unique biochemical or physical signal to attract larvae during settlement. The highest levels of early settlement were induced by *G. marina*, but not the largest initial juvenile growth, indicating that the signal for a good place for initial settlement may be different from a general nutritive signal. A *Grammatophora* species co-occurs with a natural population of *P. dumerilii* in the Bay of Naples, Italy, in conjunction with a *Posidonia oceanica* seagrass bed habitat [22,61]. A previous study of the feeding ecology of this *P. dumerilii* population found that *Grammatophora* sp. was one of the species found frequently in the benthic microalgal community of the seagrass and was also one of the most abundant genera of diatoms found in the faecal pellets of adult *P. dumerilii* collected from this habitat [22]. Considering that the laboratory population of *P. dumerilii* used in this study was predominantly sourced originally from the Bay of Naples, *G. marina* may represent an ecologically relevant cue for *P. dumerilii* larval settlement, although we cannot confirm if the *Grammatophora* sp. described in [22] was indeed *G. marina*. The physical structure of *G. marina*, as a branching diatom, may also contribute to its attractiveness to *P. dumerilii*. As young juveniles, these worms prefer to feed on erect filamentous algae due to the shape and position of their jaws [22,23].

Although many marine bacterial species in biofilm act as a larval settlement inducer, our study suggests that *P. dumerilii* does not follow the same trend, with no significant settlement induced by bacterial biofilms in the absence of microalgae. This is surprising, as many previous studies have identified specific bacterial species from marine biofilm as inducers of larval settlement, including in other polychaete species [25,33]. For example, *Pseudomonas marina*-induced settlement and metamorphosis in the spirorbid polychaete *Janua brasiliensis* [62] and *Pseudoalteromonas luteoviolacea* [30,63,64], as well as *Cellulophaga lytica*, *Bacillus aquimaris* and *Staphylococcus warneri* [29,33,60], are all positive cues for metamorphosis in the polychaete *Hydroides elegans*. The *Pseudoalteromonas* genera, in particular, is a common inducer of larval settlement across multiple marine invertebrate phyla [65]. Although we tested a *Pseudoalteromonas* species in our settlement assays with single-species bacterial biofilms, this did not induce significant *P. dumerilii* settlement over 48 h. We do not rule out that bacteria may also play a minor role in the induction of *P. dumerilii* settlement, either directly or indirectly within a mixed biofilm—they can be important as initial colonizers of a surface to establish biofilm or can contribute to biofilm robustness [66]. In addition, there may still be a bacterial species that plays a major role in *P. dumerilii* settlement that we have not yet identified through our laboratory culture-based study. Our current findings indicate that diatoms are a more important cue than bacteria for *P. dumerilii* settlement, similar to larval settlement in some bryozoans, abalone and barnacles [44,52,67]. These species-specific differences in inductive cue specificity highlight the vast diversity that exists in the mechanisms that can trigger marine invertebrate larval settlement.

### 4.2. Larval development and *Platynereis*–microalgae interactions

It is clear that in the case of *P. dumerilii*, the cue for larval settlement is a signal that indicates the presence of food for the settled juvenile. Diatoms are an important dietary component for both juvenile and adult *Platynereis* species [22,68]. Settlement assays with larvae of different ages indicate that *P. dumerilii* develop the initial capacity to respond to a *G. marina* biofilm, also known as larval competence, between 3 and 4 days old. This timing coincides with the development of the digestive tract, which is formed by the end of day 4, while food intake usually begins between 5 and 7 days of age [18,69,70]. A distinguishing feature of *P. dumerilii* development between 3 and 4 days is the formation of additional sensory organs—tentacular cirri, which emerge from the lateral head and posterior, and antennal stubs, which grow on the anterior head [69]. These sensory appendages may, therefore, be important for the detection of biofilms during settlement. Supporting this possibility, a study of the response of neuronal cells of different sensory head appendages in 6-day-old *P. dumerilii* found that cells of the cirri, and especially the antennae, were responsive to glutamate, an amino acid and sucrose, a sugar, indicative of a chemosensory role [71]. If the antennae and cirri are already functional in younger larvae, it could be these which are responsible for microalgal biofilm cue detection during *P. dumerilii* larval settlement. Previous investigations of diverse polychaete species, including *Capitella teleta*, *Hydroides elegans*, *Phragmatopoma lapidosa* and *Phragmatopoma californica* [72–75], similarly indicate that the sensory cells responsible for selecting settlement sites may be located around the mouth, pharyngeal or head sensory appendages, rather than the larval apical sensory organ, which is already present in the earlier trochophore larval stage of development.

When 2-day-old *P. dumerilii* trochophore larvae are exposed to a *G. marina* biofilm, they fail to respond, and even show very low levels of larval settlement after exposure for 48 h, by which time they are 4 days old and usually competent to settle. Similarly, premature exposure of some mollusc larvae to their preferred settlement cue can result in desensitization of the larvae and a lack of settlement response [76–79]. This is thought to be due to habituation of the chemosensory receptors that detect the cue [80]. This habituation effect provides a possible explanation for the lack of response of *P. dumerilii* to a microalgal biofilm when they encounter it prior to 3 days of age. The increased speed of response shown by *P. dumerilii* to biofilm as they age from 3 to 8 days also suggests an accumulation of receptors sensitive to microalgal biofilm during development.

For *P. dumerilii*, food is an essential requirement for growth and development beyond the three-segmented nectochaete larval stage. In the absence of food, larvae fail to add new posterior segments, and cannot complete the cephalic metamorphosis that occurs in five–six segmented juveniles [70]. The nutritional profile of a microalga may therefore also be an important factor that determines its attractiveness to a *P. dumerilii* larva or recently settled juvenile. Different diatoms and other microalgal species can contain significantly different compositions of lipid, protein and sugars [55], which may act as a unique identifier to their consumers. We also note that some diatoms are known for the secretion of noxious chemicals to deter their predators [81]. However, no microalgal species tested caused significantly higher rates of death in *P. dumerilii* larvae compared with the negative control, which would have indicated a strong toxic effect.

### 4.3. Summary and outlook

We conclude that in larval settlement, *P. dumerilii* is a generalist and will settle in the presence of any marine benthic biofilms that contain microalgae, particularly diatoms, which represent a future food source. However, the settlement decision is more robust and can occur earlier in development if the larvae receive signals from specific favoured species of diatom and coccolithophore, such as *G. marina*, some *Achnanthes* spp., *Nitzschia* spp. and the coccolithophore *Chrysotila lamellosa*, with preferred species being those known to produce EPS. This knowledge allows us to begin investigation of the link between the settlement-inducing environmental microalgal cues and the downstream internal molecular and nervous system signalling that governs settlement decisions in the *P. dumerilii* larva. The widespread occurrence of diatoms as a settlement cue for many invertebrate larvae, particularly those relevant to aquaculture, suggests that any findings on the signalling pathways of *P. dumerilii* settlement will also have relevance for these species.

In future, testing of EPS extracts of microalgal diatoms could confirm whether the settlement cue detected by *P. dumerilii* larvae is indeed a component of the EPS, and help identify the molecular nature of the cue. Mass spectrometry measurements of the nutritional profiles of the species from this study that were effective in inducing larval settlement and subsequent growth would inform on the contribution of different bioactive compounds, including vitamins, pigments, polyphenolic compounds, polyunsaturated fatty acids (PUFAs), polysaccharides, essential minerals, enzymes and peptides [82]. Further investigation of larval–diatom interactions could be facilitated by the possibility of genome editing technologies in diatoms [83]. This would allow more detailed investigations of the exact nature of the inductive molecules detected in diatoms by *P. dumerilii* larvae. For example, a specific metabolic pathway could be inhibited in a diatom biofilm, which would then be tested in settlement assays to determine whether this altered the larval settlement activity.

As the incorporation of diatoms into *P. dumerilii* culture can enhance settlement and subsequent growth, we recommend the inclusion of a select diatom species into their routine culture, and to commence administration of diatoms earlier than one week of age. This addition to *P. dumerilii* laboratory culture facility practice will help not only to enhance larval recruitment but will contribute to reducing the overall generation time of *P. dumerilii*, an important factor for the efficient establishment of mutant lines in this research model.

## Data Availability

Data and relevant code for this research work are stored in Github: [84] and have been archived within the Zenodo repository: [85]. Additional information and samples of raw images are available in the online supplementary material [86].
